# Seeking arrangements: cell contact as a cleavage-stage biomarker

**DOI:** 10.1016/j.rbmo.2023.103654

**Published:** 2024-03

**Authors:** Chloe He, Neringa Karpavičiūtė, Rishabh Hariharan, Lilly Lees, Céline Jacques, Timothy Ferrand, Jérôme Chambost, Koen Wouters, Jonas Malmsten, Ryan Miller, Nikica Zaninovic, Francisco Vasconcelos, Cristina Hickman

**Affiliations:** a Wellcome/EPSRC Centre for Interventional and Surgical Sciences, University College London 43-45 Foley St, London, W1W 7TY, UK.; b Department of Computer Science, University College London, 66–72 Gower St, London WC1E 6EA, UK.; c AI Team, Apricity, 14 Grays Inn Rd, London WC1 X 8HN, UK.; d Apricity, 13 Rue Paul Valéry, 75116 Paris, France.; e Brussels IVF, University Hospital Brussels, Jette Bldg R, Laarbeeklaan 101 1090 Jette, Belgium, Brussels.; f Ronald O Perelman and Claudia Cohen Center for Reproductive Medicine, Weill Cornell Medicine, 1305 York Ave 6th floor, New York, NY 10021, USA.; g Institute of Reproductive and Developmental Biology, Imperial College London, Hammersmith Campus, Du Cane Road, London, W12 0HS, UK.

**Keywords:** 3D reconstruction, Cleavage stage, Embryology, Cell arrangement, Cell contact, Network analysis

## Abstract

**Research question:**

What can three-dimensional cell contact networks tell us about the developmental potential of cleavage-stage human embryos?

**Design:**

This pilot study was a retrospective analysis of two Embryoscope imaging datasets from two clinics. An artificial intelligence system was used to reconstruct the three-dimensional structure of embryos from 11-plane focal stacks. Networks of cell contacts were extracted from the resulting embryo three-dimensional models and each embryo's mean contacts per cell was computed. Unpaired t-tests and receiver operating characteristic curve analysis were used to statistically analyse mean cell contact outcomes. Cell contact networks from different embryos were compared with identical embryos with similar cell arrangements.

**Results:**

At t4, a higher mean number of contacts per cell was associated with greater rates of blastulation and blastocyst quality. No associations were found with biochemical pregnancy, live birth, miscarriage or ploidy. At t8, a higher mean number of contacts was associated with increased blastocyst quality, biochemical pregnancy and live birth. No associations were found with miscarriage or aneuploidy. Mean contacts at t4 weakly correlated with those at t8. Four-cell embryos fell into nine distinct cell arrangements; the five most common accounted for 97% of embryos. Eight-cell embryos, however, displayed a greater degree of variation with 59 distinct cell arrangements.

**Conclusions:**

Evidence is provided for the clinical relevance of cleavage-stage cell arrangement in the human preimplantation embryo beyond the four-cell stage, which may improve selection techniques for day-3 transfers. This pilot study provides a strong case for further investigation into spatial biomarkers and three-dimensional morphokinetics.

## Highlights

• Increased cell contact is associated with greater developmental potential in embryos.
• This association is strongest at the eight-cell stage.
• Three-dimensional embryo analysis may provide new insights into embryo development.

## Graphical abstract

**Figure:**
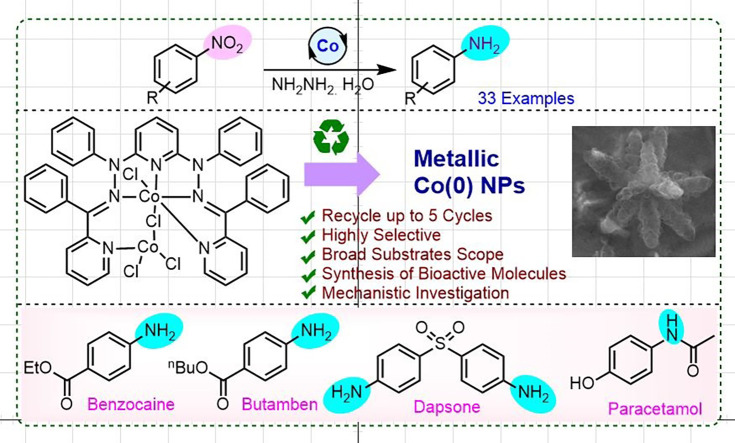

## Introduction

In the earliest stages of life, communication is key; nowhere is this more evident than in the world of embryonic development. Across many biological systems, the establishment of cellular polarity, which, in the human embryo takes place between the eight- and 16-cell stages, depends on cell-to-cell communication via intercellular contacts (Ajduk and Zernicka-Goetz, 2016). The amount of contact between blastomeres is determined by the spatial arrangement of blastomeres within the embryo, to which several factors, including cleavage patterns (Ebner et al., 2012; Cauffman et al., 2014; Ajduk and Zernicka-Goetz, 2016; Desai and Gill, 2019), fragmentation (Alikani et al., 1999a) and ploidy (Moayeri et al., 2008) may contribute.

In a clinical setting, the assessment of four- and eight-cell embryos is relatively commonplace, especially in clinics that carry out cleavage-stage transfers. Typical criteria for a cleavage-stage embryo to be considered for transfer include a good number of evenly sized blastomeres, a good degree of symmetrical division and minimal fragmentation (Rijnders and Jansen, 1998; Dennis et al., 2006; Moayeri et al., 2008). Many studies have specifically assessed cell arrangement at the four-cell stage, with most agreeing that embryos with tetrahedral cell arrangements have greater developmental potential than those without (Ebner et al., 2012; 2017; Paternot et al., 2012; Cauffman et al., 2014; Desai and Gill, 2019). To our knowledge, however, only one study has investigated cell arrangement and contact in cleavage-stage embryos beyond the four-cell stage in a clinical setting (Paternot et al., 2012). By modelling blastomeres in embryos as spheres, the investigators found an association between culture conditions, the number of cell contacts and the total surface area of contact in day-3 embryos.

This paucity of research stems from a key difficulty: visualizing embryos with many blastomeres in three dimensions. All previous work in four-cell embryo cell arrangement has involved human assessment of embryos; such an approach is challenging with eight cells owing to the exponential increase in possible contacts between blastomeres to keep track of. Moreover, although the use of confocal microscopy for understanding three-dimensional structure is standard practice in basic research, their use is infeasible in clinical practice because of concerns around staining, phototoxicity and cost (Iyer et al., 2021). Recently, He et al. (2022) described preliminary work towards a method for recovering the three-dimensional structure of cleavage-stage embryos using an artificial intelligence system on focal stacks obtained using the Hoffman modulation contrast microscopes widely integrated into modern time-lapse incubators. Their approach involves automatically detecting individual blastomeres and using the detected outlines to generate three-dimensional meshes for each blastomere in an embryo.

In this pilot study, an artificial intelligence system proposed by He et al. (2022) is used to investigate the effect of cell arrangement on clinical cleavage-stage human embryos at the four- and eight-cell stages. Networks of blastomere contacts are derived from three-dimensional visualizations of embryos and perform simple network analyses. From these analyses, we propose a simple, autonomously assessable quantitative biomarker for the developmental potential of clinical human embryos: the mean number of intercellular contacts per blastomere.

## Materials and methods

### Study population, setting and clinical data collection

This pilot study was a retrospective analysis of two imaging datasets from two different clinics. All data were captured at 11 focal planes on Embryoscope incubators between 2018 and 2020 and standardized using the Super-Focus method (He et al., 2022).

The first dataset (DS1) consisted of 303 t4 embryos from the first clinic with information on blastulation (blastulated and non-blastulated) and Gardner grade (Gardner and Schoolcraft, 1999). The second dataset (DS2) consisted of 217 embryos from the second clinic at t4 and t8 with information on Gardner grade, biochemical pregnancy, live birth, kinetics (annotated by embryologists) and preimplantation genetic testing for aneuploidy (PGT-A) (which was conducted in-house on all embryos on day 5).

The study was approved by HRA and Health and Care Research Wales (HCRW) on 12 January 2021 (IRAS 287428).

### Three-dimensional cell contact analysis

Three-dimensional reconstructions were obtained from each focal stack using the system proposed by He et al. (2022). The reconstructions were visualized in the Unity game engine (v2021.3.7f1) and manually validated with incorrect reconstructions being excluded. After manual validation, DS1 was reduced to 238 four-cell embryos. DS2 was reduced to 115 eight-cell embryos and 201 four-cell embryos owing to factors such as the embryos being too compacted for cell boundaries to be visible, cells not being detected, excessive fragmentation and the embryo being cut off by the well. A total of 101 embryos had reconstructions for both the four- and eight-cell stages. Further details on the datasets after manual validation are presented in Table 1.

**TABLE 1:** THE MANUALLY VALIDATED DATASET

Dataset	Stage	Parameter	*n*
DS1	t4		238
		Blastulation (blastulated/non-blastulated)	203/35
		Blastocyst quality (good/poor)	106/97
DS2	t4		201
		Blastocyst quality (good/poor/unknown)	125/75/1
		Blastocyst transfers	131
		Biochemical pregnancies	92
		Live births	68
		Miscarriages	11
		Ploidy (euploid/aneuploid)	151/50
	t8		115
		Blastocyst quality (good/poor)	74/41
		Blastocyst transfers	80
		Biochemical pregnancies	59
		Live births	44
		Miscarriages	7
		Ploidy (euploid/aneuploid)	88/27

**Figure 1:**
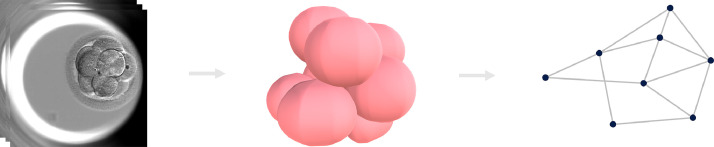
The three-dimensional analysis process: focal stacks (left) were used to generate three-dimensional models of embryos (middle) from which cell contact networks (right) were obtained.

The existence of cell contacts was determined by scaling the meshes of each blastomere by a small factor (in our case, 1.05, determined empirically over a subset of 10 embryos) and detecting the presence of any resulting overlap. From these calculations, networks of cell contacts were constructed for each embryo, with nodes in the networks representing each blastomere and links between nodes representing cell contacts between the linked nodes. The cell contact networks were then assessed for the mean number of cell contacts per blastomere. An overview of the process is presented in Figure 1. The reconstruction process was carried out on a server running Ubuntu 20.04 machine with an Intel i7-9700K CPU, 64 GB RAM and an NVIDIA Titan X GPU. A Windows 10 desktop machine with an Intel i3-6100 CPU and 36 GB RAM were used for all visualization and further analysis.

### Characterization of cell arrangement

The cell arrangement in each embryo was characterized using Hickman vectors, a mathematical representation of cell contact originally developed for the characterization of four-cell embryos (Hickman et al., 2021). A Hickman vector representing an *N*-cell embryo is a list of numbers in which the *n*th number indicates the number of blastomeres with exactly *n* cell contacts (for 0≤n <N). The frequency distribution of the Hickman vectors observed across the datasets was computed.

The Pandas (v1.4.1) package in Python (v3.10.3) was used for all analyses.

### Statistical analysis

Unpaired t-tests were used to compare the mean number of cell contacts per blastomere with blastulation, blastocyst quality, biochemical pregnancy and live birth outcomes. Blastocyst quality was measured as a binary variable (good/poor) derived from the Gardner grade. A ‘good’ embryo was defined as having a Gardner grade with EXP>2, ICM>C and TE>C. Analyses of biochemical pregnancy, live birth and miscarriage rates only included transferred embryos (here, miscarriage is defined as the loss of a clinical pregnancy within the first 20 weeks). The Spearman rank correlation coefficient was used to evaluate the association between the mean number of cell contacts per blastomere at the four- and eight-cell stages for a given embryo.

All statistical analysis was carried out using the Pandas (v1.4.1) and Correlation (v1.0.0) packages in Python (v3.10.3). Correlation coefficient confidence intervals were computed using the bootstrapping method implemented in the Correlation (v1.0.0) package with 10000 samples at 95% confidence.

### Evaluation of predictive power

The power of the mean number of contacts per blastomere as a predictor of biochemical pregnancy and live birth was evaluated using five-fold cross-validation on eight-cell embryos in DS2. For each fold, a simple threshold-based model was fitted to the training folds and evaluated on the held-out fold using the accuracy, precision, sensitivity, F1 and area under the receiver operating characteristic curve (AUC) metrics. The threshold-based models took the following form:

$$h\left(x\right)\;=\;\left\{ \begin{array}{c} \;positive\;outcome\;x>\theta ,\\ negative\;outcome\;other\;wise \end{array}\right.$$

where x is the input feature, h(x) is the outcome prediction based on x, and θ is a cut-off threshold to be calibrated during training. Training involved an exhaustive search of potential thresholds with the goal of maximizing AUC on the training folds. The performance of the model was benchmarked against the KIDScore D3 (Petersen et al., 2016), a well-known biomarker for cleavage-stage embryo viability based on kinetic parameters (tPNf, t2, t3, t5, t8), using the same evaluation protocol. Model AUCs were compared using unpaired t-tests.

All evaluations were carried out using the scikit-learn (v1.2.1) package in Python (v3.10.3).

## Results

Comparisons between the mean number of blastomere contacts and embryo outcome are presented in Table 2. At t4, a higher mean number of contacts per cell was associated with greater rates of blastulation in DS1 (*P* = 0.007) and blastocyst quality in both DS1 (*P* = 0.003) and DS2 (*P* = 0.014). At t8, a higher mean number of contacts was associated with increased blastocyst quality (*P* = 0.017), biochemical pregnancy (*P* = 0.003) and live birth (*P* < 0.001).

**TABLE 2:** COMPARISON OF MEAN NUMBER OF CELL CONTACTS ACROSS DIFFERENT OUTCOMES

Dataset	Stage	Comparison (outcome 1 versus outcome 2)	Outcome 1 (mean ± SD)	Outcome 2 (mean ± SD)	t-statistic	*P*-value
DS1	t4	Blastulated versus non-blastulated	2.54 ± 0.46	2.30 ± 0.61	2.713	0.007^a^
		Good versus poor blastocyst quality	2.61 ± 0.40	2.46 ± 0.52	3.046	0.003^a^
DS2	t4	Good versus poor blastocyst quality	2.52 ± 0.45	2.35 ± 0.48	2.475	0.014^a^
		Pregnant^b^ versus not pregnant	2.49 ± 0.46	2.41 ± 0.57	0.830	0.408
		Live birth versus no live birth	2.44 ± 0.46	2.45 ± 0.55	−0.095	0.925
		Live birth versus miscarriage	2.44 ± 0.46	2.59 ± 0.44	−1.005	0.318
		Euploid versus aneuploid	2.44 ± 0.49	2.49 ± 0.42	−0.607	0.545
	t8	Good versus poor blastocyst quality	3.36 ± 0.61	3.08 ± 0.55	2.423	0.017^a^
		Pregnant^b^ versus not pregnant	3.32 ± 0.56	2.87 ± 0.63	3.041	0.003^a^
		Live birth versus no live birth	3.40 ± 0.53	2.90 ± 0.59	3.684	<0.001^a^
		Live birth versus miscarriage	3.40 ± 0.53	3.00 ± 0.50	1.842	0.072
		Euploid versus aneuploid	3.21 ± 0.60	3.44 ± 0.57	1.725	0.087

a *P* < 0.05.

b Biochemical pregnancy. DS1, dataset 1; DS2, dataset 2.

**Figure 2:**
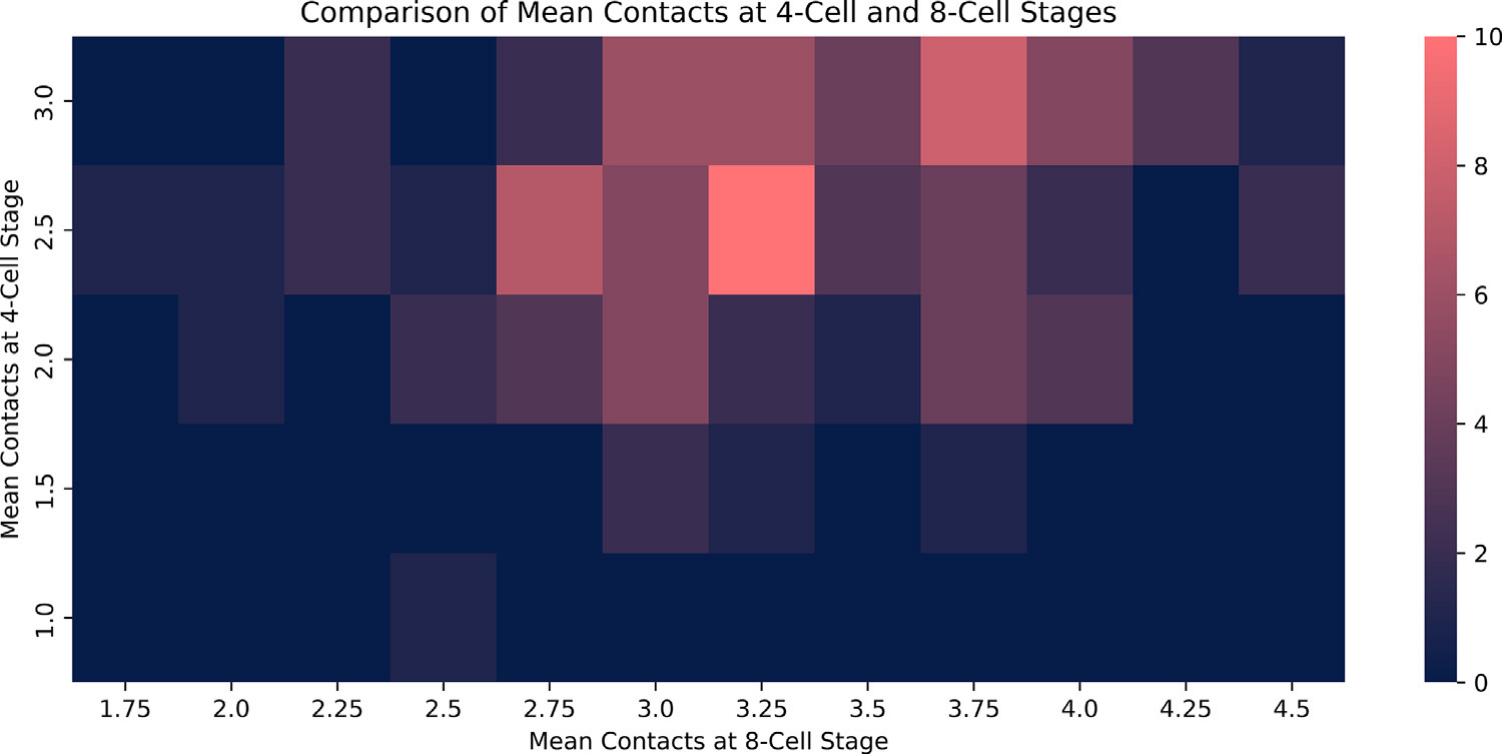
Heatmap of mean contacts at the t4 stage versus mean contacts at the t8 stage. The axes represent the number of cell contacts at the four- and eight-cell stages. The colour of each grid square represents the frequency of embryos with a specific combination of four- and eight- cell contacts. A slight upward trend can be seen.

Mean contacts at t4 weakly correlated with those at t8 (ρ = 0.24, 95% CI 0.04 to 0.42). A plot is presented in Figure 2.

**Figure 3:**
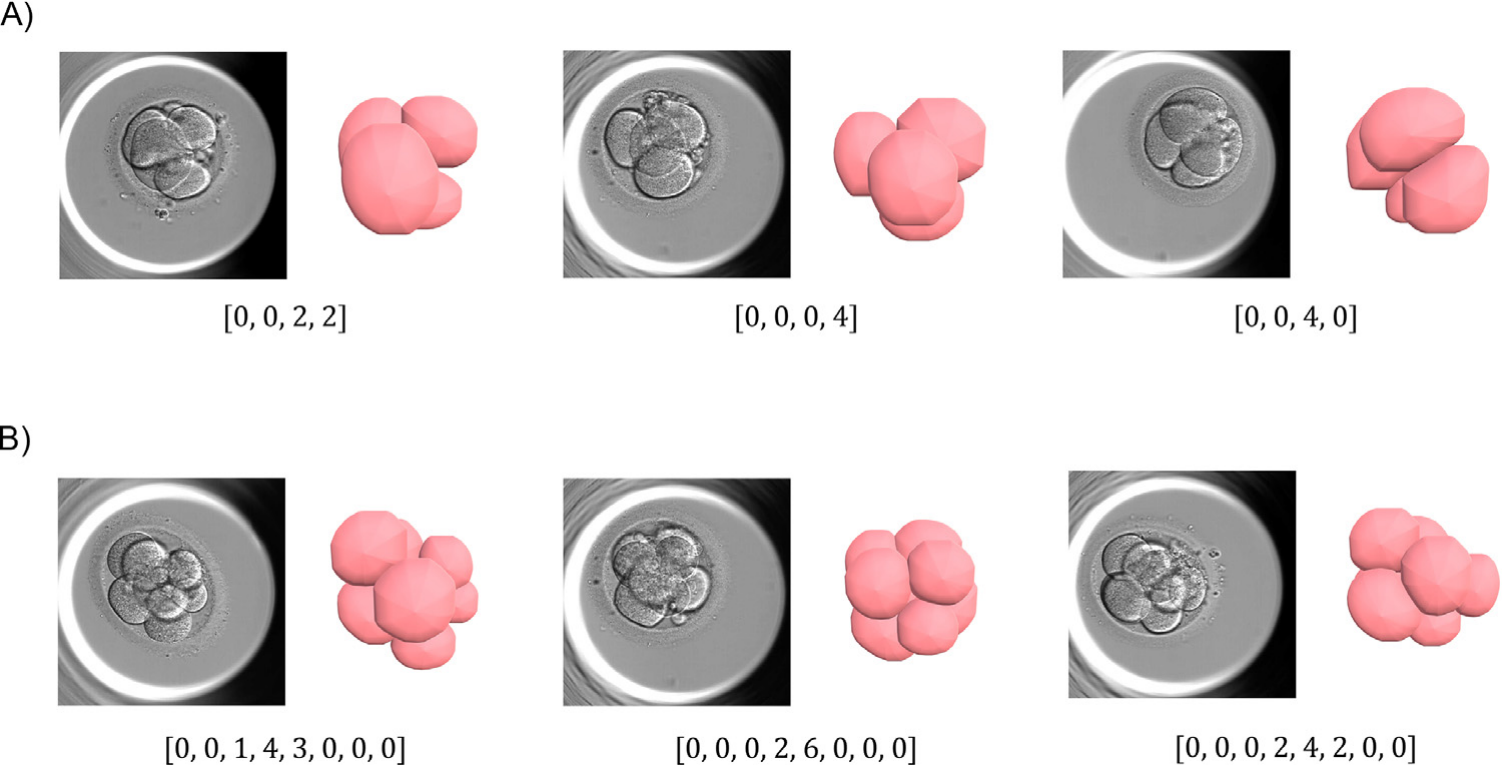
Examples of the most common cell arrangements at the (A) four-cell and (B) eight-cell stages.

At the four-cell stage, it was observed that embryos fell into nine distinct cell arrangements (Table 3). The most common of these were [0,0,2,2] (pseudotetrahedral), [0,0,0,4] (tetrahedral) and [0,0,4,0](planar). Examples of each cell arrangement can be found in Figure 3A. At the eight-cell stage, embryos displayed a great degree of variation with 59 distinct cell arrangements, the most common of these, [0,0,1,4,3,0,0,0], representing only seven embryos. Examples of the most common eight-cell arrangements are presented in Figure 3B.

**TABLE 3:** FREQUENCIES OF FOUR-CELL BLASTOMERE ARRANGEMENTS

Arrangement	Arrangement nickname	Frequency in DS1	Frequency in DS2
[0, 0, 2, 2]	Pseudotetrahedral	95	80
[0, 0, 0, 4]	Tetrahedral	86	60
[0, 0, 4, 0]	Planar	19	30
[0, 1, 2, 1]	Closed Y	17	16
[0, 2, 2, 0]	Linear	11	10
Other	NA	10	5

DS1, dataset 1; DS2, dataset 2; NA, not applicable.

A comparison of the mean number of blastomere contacts at the eight-cell stage and the KIDScore D3 as predictive markers of biochemical pregnancy and live birth can be found in Table 4. The models achieved similar AUC scores when predicting biochemical pregnancy suggesting comparable predictive power (*P* = 0.533) and our method outperformed the KIDScore D3 when predicting live birth (*P* = 0.029). For predicting biochemical pregnancy, the mean threshold values across all folds (mean ± SD) were 2.4 ± 0.8 for the KIDScore D3 and 3.2 ± 0.2 for the mean number of blastomere contacts. Similarly, for predicting live birth, the mean threshold values were 1.8 ± 0.4 for the KIDScore D3 and 2.9 ± 0.2 for the mean number of contacts.

**TABLE 4:** PERFORMANCE COMPARISON BETWEEN THE MEAN NUMBER OF CELL CONTACTS AND KIDSCORE D3 AS PREDICTORS OF BIOCHEMICAL PREGNANCY AND LIVE BIRTH

Outcome	Metric	Mean cell contacts	KIDScore D3
Biochemical pregnancy	Accuracy	0.59 ± 0.11	0.74 ± 0.08
	Precision	0.89 ± 0.14	0.77 ± 0.06
	Sensitivity	0.52 ± 0.13	0.91 ± 0.11
	F1	0.65 ± 0.11	0.83 ± 0.06
	AUC	0.64 ± 0.18	0.58 ± 0.10
Live birth	Accuracy	0.68 ± 0.08	0.64 ± 0.08
	Precision	0.73 ± 0.04	0.63 ± 0.05
	Sensitivity	0.74 ± 0.20	0.95 ± 0.10
	F1	0.72 ± 0.13	0.76 ± 0.07
	AUC	0.65 ± 0.06	0.54 ± 0.07

All metrics were evaluated on dataset 2 (D3) blastocysts transferred on day 5 after preimplantation genetic testing for aneuploidy. Values are shown as mean ± SD for all folds in the five-fold cross validation.

AUC, area under the receiver operating characteristic curve; F1, the harmonic mean of the precision and sensitivity.

## Discussion

The results of our study echo findings in previous studies that a greater degree of cell contact in the four-cell embryo is conducive to greater developmental potential. We further demonstrated that these findings translate to eight-cell embryos. In this section, we first compare our study to previous works before examining our findings from biological perspective. We then discuss the novel computational aspects of our work followed by the limitations of this study.

### Comparison with previous studies on cell arrangement

#### Four-cell arrangement

Previous studies (Ebner et al., 2012; 2017; Cauffman et al., 2014; Paternot et al., 2014; Desai and Gill, 2019) on the arrangement of blastomeres in four-cell human embryos manually assessed cell arrangement by eye. Our study, in contrast, used a computational method. The advantages of taking this approach include faster and more scalable assessment as well as a reduction in the inter- and intra-observer variability inherent in manual embryological annotation (Baxter Bendus et al., 2006; Khosravi et al., 2019). Notwithstanding, the results of our computational analysis strengthen the argument that increased cell contact in four-cell embryos is associated with greater developmental potential.

#### Eight-cell arrangement

At the eight-cell stage, an increase in the number of blastomeres present, as well as the possible interactions between them, rule out the feasibility of quick manual assessment. To address this, Paternot et al. (2012) used a semi-automated approach, manually labelling the diameters of blastomeres and using these annotations to generate spheres representing each blastomere. Cell contacts were obtained by detecting overlapping spheres. Although similar in concept, the system used in our work takes a more complex approach, making use of automatically generated cell membrane annotations to represent blastomeres as opposed to spherical approximations. This allowed for a more complex modelling approach which may explain why we detected a correlation between eight-cell stage blastomere contacts and live birth, whereas Paternot et al. (2012) did not.

### Characterization of blastomere arrangement in embryos

Over the past years, several systems for the characterization of blastomere arrangement in four-cell embryos have been forwarded. Among these are the tetrahedral/non-tetrahedral system (Desai and Gill, 2019), the tetrahedral/planar system (Ebner et al., 2012; 2017; Milewski and Ajduk, 2017) and Hickman vectors (Hickman et al., 2021). Such systems are useful as they provide a standardized way to categorize and communicate relevant information about embryos encountered in clinical practice, and can thus simplify clinical decision-making.

To our knowledge, no studies have attempted to characterize blastomere arrangement in eight-cell embryos. While simply extending Hickman's system to eight-cell embryos may seem a straightforward approach to fill this gap, our results demonstrate that the large increase in possible blastomere arrangements at the eight-cell stage preclude this: the system was so fine-grained that even the largest group consisted of too few embryos to draw conclusions on clinical outcomes in relation to group membership or the relative prevalence of different groups. Our work overcame this issue by condensing networks into a single numerical descriptor corresponding to the mean degree across all nodes. Although straightforward, such an approach proved sufficient to achieve, albeit with several major caveats discussed in the Limitations section, predictive performance comparable to a well-established existing method based on multiple kinetic parameters. Future works may investigate the use of cluster analysis and graph embedding techniques to identify more coarse-grained, clinically usable characterizations of eight-cell embryos.

### Blastomere contact and developmental potential

The development of an embryo into a blastocyst depends on a complex series of events that involve the interaction of multiple signalling pathways, genetic and epigenetic factors, and physical properties of the embryo. Our results suggest that more contact between the blastomeres throughout early embryo development is associated with greater developmental potential.

One explanation for this association may be that a greater degree of blastomere contact enables greater communication between blastomeres. Many of the proteins involved in cell adhesion serve a dual role in enabling cell signalling (Klezovitch and Vasioukhin, 2015; Shawky and Davidson, 2015). Cell signalling is especially important for self-organization and fate determination in the early embryo (Andrzej and Tarkowski, 1959; Rossant, 1976; Strumpf et al., 2005; Ralston et al., 2010; Klezovitch and Vasioukhin, 2015; Saiz et al., 2016; Menchero et al., 2019). It can, therefore, be reasoned that greater levels of contact facilitates improved communication and thus greater developmental potential. Indeed, this notion forms part of the rationale behind the practice of embryo defragmentation, as fragments often interfere with cell junctions (Keltz et al., 2006). Another possible explanation may stem from the fact that early compaction is associated with activation of the embryonic genome and greater developmental potential (Desai et al., 2000; Öztürk et al., 2022; Skiadas et al., 2006). More blastomere contact may, therefore, as opposed to being a causal factor, just be a marker for greater levels of compaction and thus the transcription and activation of the embryonic genome that increases an embryo's developmental potential.

We also observed stronger associations between improved outcomes and blastomere contact at the eight-cell stage than the four-cell stage. This may be explained by the fact that totipotency is gradually lost from four-cell stage onwards as lineage transcription factors change gene expression across the different blastomeres, eventually committing them to different developmental fates (Tarkowski and Wróblewska, 1967; Strumpf et al., 2005; Ralston et al., 2010; de Paepe et al., 2014; Wicklow et al., 2014; Goolam et al., 2016). By the eight-cell stage, this process is more advanced, and thus arguably more representative of the embryo at transfer and implantation. In contrast, four-cell stage embryos are still totipotent and may rely more heavily on maternal mRNA.

It has also been suggested that cell arrangement in an embryo may reflect the presence of cleavage anomalies at the four-cell stage (Piotrowska-Nitsche and Zernicka-Goetz, 2005; Ebner et al., 2012; Ajduk and Zernicka-Goetz, 2016). It is, however, unclear how this may translate to the eight-cell stage and the multitude of possible arrangements we identified. Additional research efforts are necessary to draw any definitive conclusions.

### Representation matters

The past few years have seen a surge of interest in the use of artificial intelligence tools for embryo selection. Many recent studies have been based on image analysis techniques from computer vision, with a great number of these trying to predict embryo viability directly from images and videos (Khosravi et al., 2019; Ver Milyea et al., 2021; Berntsen et al., 2022; Kragh et al., 2022). A key difference between the present study and previous studies is that we operate on cell contact networks derived from embryo images as opposed to the images themselves. Taking such an approach carries an important advantage: it gives us a representation of cell organization in an embryo that is ‘invariant’ with respect to the embryo's orientation.

**Figure 4:**
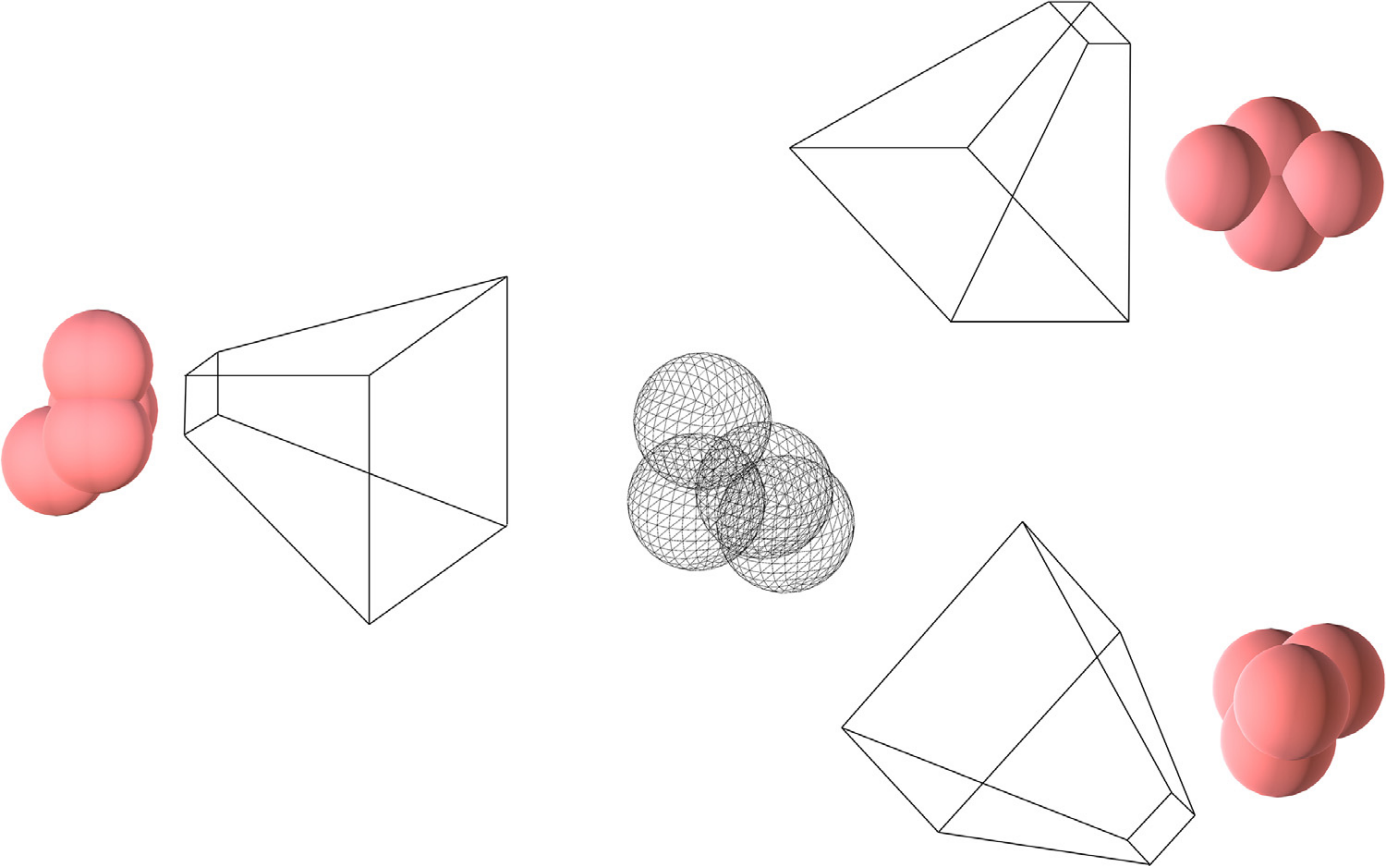
A three-dimensional model of an embryo (shown as a wireframe) viewed from different angles (illustrated using frustums). The resulting view from each angle is shown next to its respective frustrum.

Roughly speaking, ‘orientation invariance’ refers to the fact that even when an embryo is orientated differently under a microscope, its representation as a cell contact network remains the same. This cannot be said about representing embryo cell arrangement using raw image data: the same embryo orientated in different ways will yield different focal stacks (Figure 4). As a result, when training computer vision systems, a large quantity of data is needed to establish that the different focal stacks correspond to the same arrangement, a problem that existing data augmentation methods cannot overcome.

Moreover, our approach explicitly encodes the ideas that a cell affects the other cells in its neighbourhood and that the overall spatial arrangement of the embryo is of importance. Representing embryos as cell contact networks can thus provide a more biologically grounded basis for automated embryo assessment than purely image-based works. Future works may augment the nodes with further information derived from the embryo, such as features derived from analyses of blastomere shape and cytoplasm texture to produce predictive models for clinical outcomes. They may also integrate temporal information to understand how interactions between cells change over time, giving rise to a new field of three-dimensional morphokinetics.

### Limitations

The biggest limitation of our study lies in the data used: this was a small-scale analysis of two very different embryo datasets from two clinics. This limitation stems from the need for manual validation of reconstructions given the newness of the technique as well as the resource constraints of a pilot study. DS2 harbours several biases as it was collected with a view to maximizing data on transferred embryos and PGT-A. It is, therefore, not a representative sample of the general embryo population, which is not transferred. In addition, the fact that PGT-A was conducted on all DS2 embryos necessarily implies they survived to blastocyst stage, which creates a survivorship bias against embryos that arrest in the cleavage or morula stages.

Furthermore, the applicability of our findings to cleavage stage transfers is limited as the study comprised only embryos that were cultured to and transferred at the blastocyst stage. It is, therefore, possible that the associations observed in our study may not hold true for embryos transferred on day 3. A similar limitation is important to consider when reviewing the comparison between our method and the KIDScore D3. While our classifier was trained to predict biochemical pregnancy after PGT-A and blastocyst transfer, the KIDScore D3 was trained to predict implantation from cleavage-stage transfers. Both algorithms, however, were tested on the former population, which raises the possibility that results might diverge when applied to day-3 transfers. As a result, larger collaborative studies with more embryos and appropriate end points are needed to comprehensively evaluate the utility of the method proposed here.

Finally, the three-dimensional reconstruction system was not applicable to all cleavage-stage embryos, especially those obscured by the edge of the well, severely fragmented or having undergone substantial compaction. Nevertheless, a failure to carry out three-dimensional reconstruction does not automatically indicate that nothing can be learnt about the embryo, especially in the latter two cases. As previously mentioned, early compaction has been associated with greater developmental potential (Aslan Öztürk et al., 2022; Skiadas et al., 2006). Moreover, in theory, these embryos have the most blastomere contact of all; therefore, the exclusion of these embryos may mean that we underestimated the strength of the relationship between cell contact and developmental potential (Skiadas et al., 2006). Similarly, severe fragmentation has been associated with negative clinical outcomes, including lower implantation rates (Alikani et al., 1999b; Keltz et al., 2006). Future studies may explore the potential for automated detection of these conditions before attempting reconstruction; however, our findings suggest that, in most cases falling outside these two extremes, three-dimensional analysis has the potential to be a valuable tool.

In conclusion, in this pilot study, we have provided evidence to support the clinical significance of cleavage-stage cell arrangement in the human preimplantation embryo extending beyond the four-cell stage. Our results demonstrate significant correlations between the amount of cell contact in an embryo and developmental outcomes, with the strongest associations seen in eight-cell embryos. Moreover, we highlight that cell contact at the eight-cell stage may be competitive with more traditional morphokinetic scoring algorithms when predicting biochemical pregnancy and live birth, which could be helpful for improving embryo selection and cycle planning, particularly in situations in which a day-3 transfer may be necessary. Overall, our findings suggest that three-dimensional morphokinetics may represent a promising avenue for further investigation.

## Data Availability

The data that has been used is confidential.
